# Endovascular Treatment of Aortic Stump Rupture After Extra-anatomic Aortoduodenal Fistula Repair is not a Definitive Treatment: A Case Report and Literature Review

**DOI:** 10.1016/j.ejvsvf.2022.03.004

**Published:** 2022-03-30

**Authors:** Elise Beijer, Vincent P.W. Scholtes, J. Hillian Nederhoed, Rutger J. Lely, Arjan W.J. Hoksbergen

**Affiliations:** a Department of Vascular Surgery, Amsterdam University Medical Centre, VUmc, Amsterdam, the Netherlands; b Department of Radiology, Amsterdam University Medical Centre, VUmc, Amsterdam, the Netherlands

**Keywords:** Aortoduodenal fistula, Aortic stump blow-out, Aortic stump rupture, Endovascular treatment, Amplatzer® Vascular Plug, ABFB, Axillo-bifemoral bypass, ADF, Aortoduodenal fistula, AVP, Amplatzer® Vascular Plug, FDG, Fluor-18-glucose, ICU, Intensive Care Unit, PET, Positron Emission Tomography

## Abstract

**Introduction:**

Endovascular treatment of an aortic stump rupture is technically feasible. Whether this is a definitive treatment or a bridge to further surgery is unknown.

**Report:**

Previously a Case of an aortic stump rupture following extra-anatomic repair of a recurrent aortoduodenal fistula (ADF), which was successfully treated endovascularly by placement of an Amplatzer® Vascular Plug was described. The patient survived this acute procedure, but four years later was admitted with fever and back pain. Imaging revealed progressive enlargement of the aortic stump. A re-exploration was performed with removal of the infected aortic stump including the Amplatzer plug. A new aortic stump was created together with resection of an adherent part of the duodenum. The patient was discharged after five months and was able to survive for two more years without any recurring vascular complications.

**Discussion:**

This Case demonstrates that after four years, endovascular treatment was *not* a definitive treatment for aortic stump rupture. Endovascular treatment should be followed by definitive treatment when the patient is fit for surgery, especially in cases of ADF. If the patient is unfit for surgery, conservative treatment with culture based antibiotics is a reasonable alternative. Positive obstinacy lengthened the survival of this patient with eight years of reasonably good quality life.

## Highlights

• Aortic stump ruptures are frequently lethal and demand rapid treatment.
• Aortic stump ruptures can be treated endovascularly.
• Late aortic stump enlargement prompts for definitive surgical repair.
• In certain cases, endovascular treatment should be regarded as a bridge to further surgery.

## Introduction

An aortoduodenal fistula (ADF) is a relatively rare but serious complication following open or endovascular aortic aneurysm repair.^1^ The definitive treatment consists of removing the fistula track and affected tissue, removal of the infected graft, and vascular reconstruction by either in situ repair or extra-anatomic bypass grafting with aortic stump formation.^2^, ^3^, ^4^ ADF treatment is associated with a high mortality rate and part of this is caused by aortic stump rupture or re-bleeding. An aortic stump rupture is frequently lethal and demands prompt treatment. Previously successful endovascular treatment with placement of an Amplatzer® Vascular Plug (AVP) was described.^5^ An endovascular approach was chosen because of the acute setting with severe haemodynamic shock combined with extensive previous abdominal surgery. Initially it was hoped that this endovascular treatment could be a definitive solution. However, four years later sudden pain, infection and progressive stump enlargement prompted a definitive open repair with removal of the infected stump and the AVP with creation of a neo-aortic stump. This report describes the Case and a review of existing literature on endovascularly treated aortic stump ruptures.

## Case report

**Figure 1:**
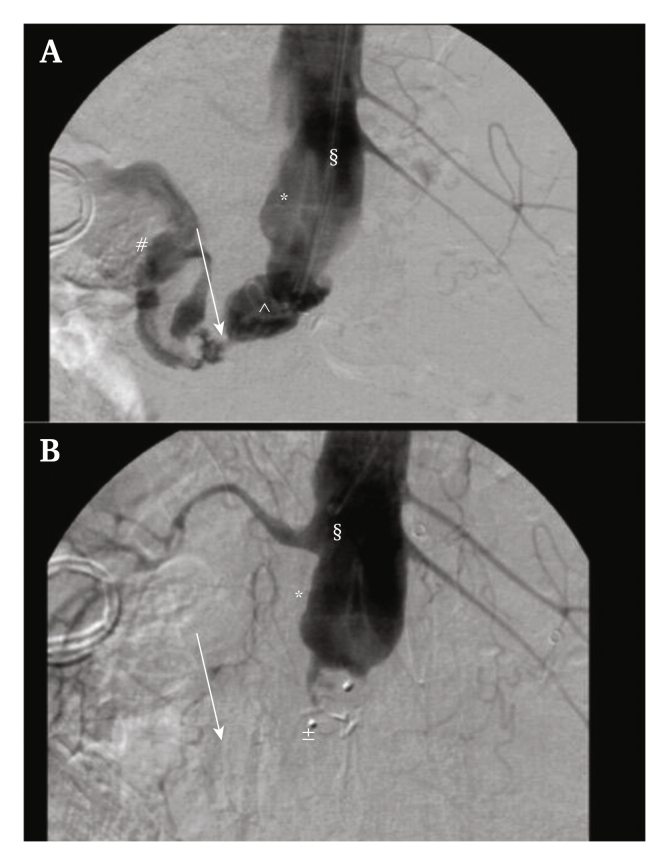
**Angiography and placement of the Amplatzer® Vascular Plug in the aortic stump**. Angiography demonstrating the aorta (∗) the duodenum (#) and the aortoduodenal fistula (↓). The upper image (A) demonstrates contrast passage from the aorta through the aortoduodenal fistula towards the duodenum. The lower graph (B), 23 minutes later, demonstrates successful closure of the aortic stump with no more contrast leakage through the aortoduodenal fistula. The angiographic catheter is marked with §, the guide wire with ˆ and the 16 mm Amplatzer® Vascular Plug with ±.

In March 2013 a 67 year old man presented with persistent duodenal leakage following recent removal and in situ repair of an infected aortic bifurcation prothesis. The patient had undergone multiple surgical procedures before transfer (Supplementary figure 1). Excision of the aortic bifurcation prosthesis, a right sided axillobifemoral extra-anatomic bypass, aortic stump formation and an 8 cm duodenal resection with gastrojejunostomy was performed. Twelve weeks later, massive haematemesis and haemodynamic instability occurred due to an aortic stump bleeding into the duodenum.^5^ The patient was admitted to the intensive care unit (ICU) and was considered unsuitable for open surgery due to severe haemodynamic instability and multiple previous abdominal operations (Supplementary figure 1). He was brought to the angiosuite and a 16 mm Amplatzer® Vascular Plug (AVP) was implanted into the defect via upper arm access. This effectively stopped the bleeding (Fig. 1). Because of a good clinical recovery, combined with a hostile abdomen (Supplementary figure 1), no further surgery was performed. Three months after the intervention he was discharged to a rehabilitation clinic in good clinical health.^3^ After a total of six months intravenous antibiotics, no clinical signs of ongoing infection were present and therefore the antibiotics were stopped. The patient was clinically evaluated every 7–8 weeks, due to his kidney drain checks and infection parameters were checked and followed.

**Figure 2:**
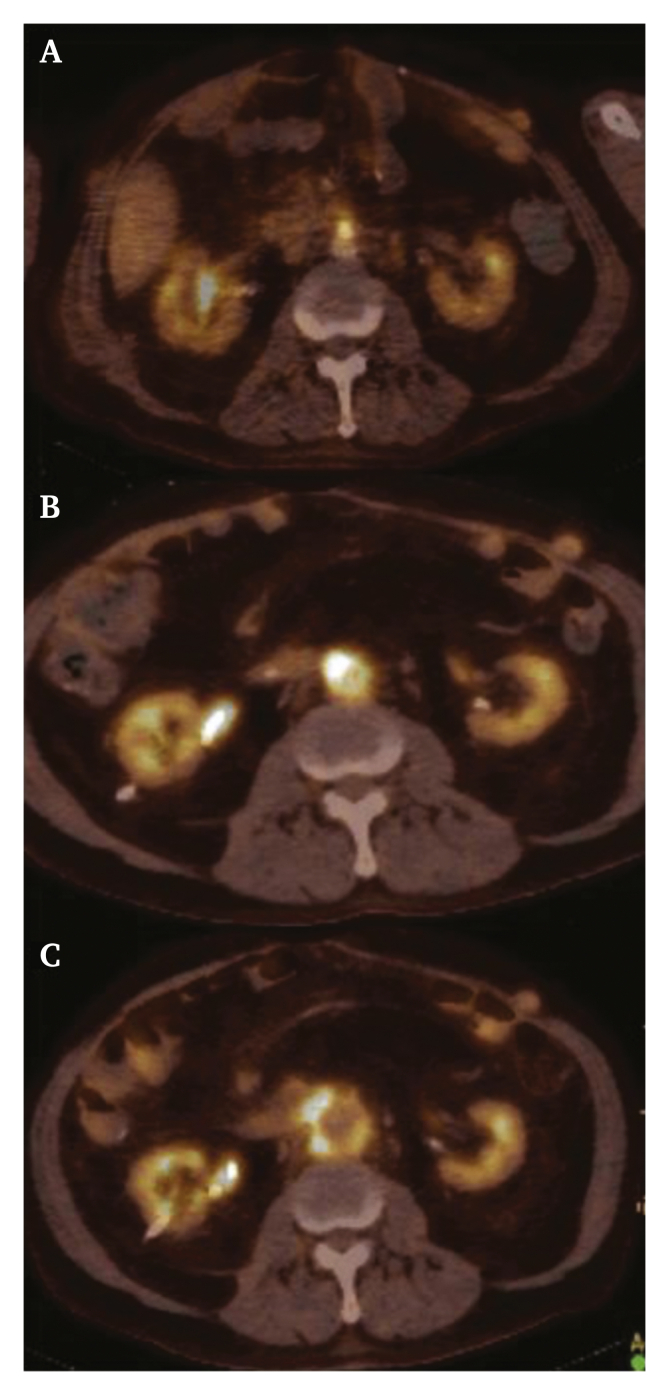
**PET scan demonstrating persistent infection and progressive dilatation of the aortic stump**. PET-scans were performed which demonstrated persistent infection of the aortic stump, with the Amplatzer® Vascular Plug in situ. Furthermore, it showed progressive dilatation of the aortic stump without signs of acute rupture. The upper image (A) shows small amounts of fluor-18-glucose (FDG) uptake around the aortic stump (diameter 20mm) in 2015 (two years after the endovascular repair). The middle image (B) shows increased FDG uptake and signs of enlargement of the aortic stump (diameter 23mm) at the beginning of January 2019. The lower image (C) demonstrates further increase in FDG uptake around the aortic stump and progressive enlargement (diameter 36mm) by the end of January 2019.

**Figure 3:**
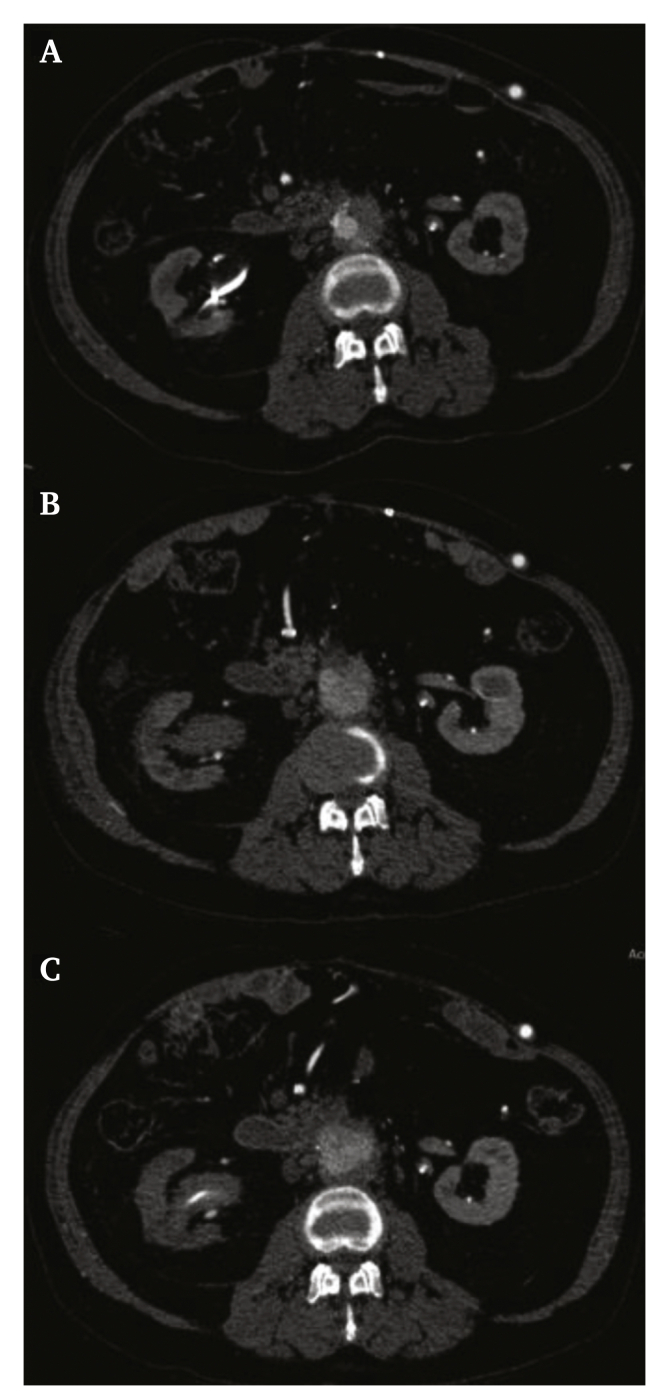
**CTA demonstrating progressive dilatation of the aortic stump**. CTA demonstrating persistent infection of the aortic stump, with the Amplatzer® Vascular Plug in situ. Also, it shows progressive dilatation of the aortic stump without signs of acute rupture. The upper, middle and lower images (A, B and C) show the progressive enlargement of the aortic stump from day 0 (A) to day 4 (B) and day 11 (C) during admission.

**Figure 4:**
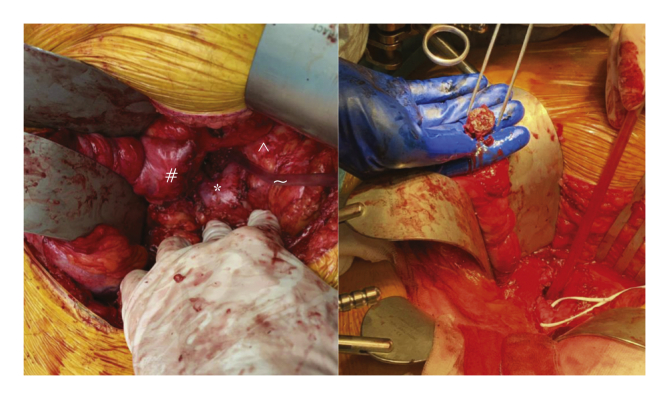
**Peri-operative images**. The left panel demonstrates the new aortic stump (∗), the duodenum (#), pancreas (ˆ) and suction instrument (∼). The right panel showing the removed Amplatzer Vascular Plug (AVP) in the surgeon's hand.

In December 2018, four years after cessation of the intravenous antibiotics, the patient was re-admitted complaining of respiratory distress, fever and signs of general discomfort. A PET scan demonstrated increased fluor-18-glucose (FDG) uptake around the aortic stump. The patient was treated for six weeks with intravenous meropenem with good clinical results after which he was discharged home. Unfortunately, two weeks later he was re-admitted with fever, discomfort, and also back pain. A PET scan and CT angiography (CTA) demonstrated persistent infection of the aortic stump with progressive dilatation of the aortic stump without signs of rupture (Figure 2, Figure 3). Because of the ongoing infection combined with progressive stump dilatation and back pain it was decided to perform a re-exploration. A right sided subcostal incision was performed with extensive adhesiolysis. Access to the aortic stump and the AVP was obtained via a partial right sided visceral rotation. The aorta was cross clamped proximal to the renal arteries and the aortic stump, old sutures, Teflon pledgets and AVP were resected. A new aortic stump was created by suturing the aorta just distally to the right renal artery with Prolene 4.0 sutures, sacrificing a left accessory renal artery (Fig. 4). A small part of the severely adherent distal duodenum including the fistula was resected as well. Due to multiple prior surgical abdominal procedures no mobile omentum was available to perform an omentoplasty. Post-operative recovery was complicated by a persisting severe gastroparesis (several months) and a partial wound dehiscence. Bacterial cultures demonstrated Enterococcus Faecium, Candida Albicans and Candida Glabrata species, for which selective treatment was started. There was no deterioration of renal function. The patient gradually recovered and could finally be discharged to a rehabilitation clinic five months post-operatively. Up to two years after presentation, the patient was in a reasonably good clinical condition without signs of complications. He died in January 2021 due to a non-related cause.

## Discussion

In this report a Case is described in which endovascular treatment of an aortic stump rupture was initially a successful life saving treatment. Due to the patient's clinical condition, ongoing conservative treatment was chosen. However, four years later the patient developed complications of a persistent aortic stump infection for which definitive open surgical reconstruction was performed.

Case reports describing endovascular treatment of aortic stump ruptures are relatively rare. Review of the literature revealed only four other cases (Supplementary Table 1). Terasaki et al.^6^ described the case of a 19 year old male patient who sustained various abdominal gunshot wounds. Following multiple open vascular interventions, the patient developed a large pseudoaneurysm of the abdominal aorta. This was treated on day eighteen by axillobifemoral bypass (ABFB) with iliac ligation and formation of an infrarenal aortic stump. Fifty days later aortic stump rupture occurred which was successfully treated with insertion of coils and gelatin sponge pledges. The patient was treated with antibiotics and no complications occurred during a limited follow up of 150 days. Marone et al.^7^ described the case of a 59 year old man who had undergone aortobifemoral bypass removal with ABFB and aortic stump formation due to an ADF. Nine years later the patient developed a second ADF with haematemesis which was treated successfully with an 18 mm AVP. However, Marone et al. decided to use the endovascular treatment as a bridge to surgery. Five days after plug placement relaparotomy was performed with plug removal, infrarenal aortic ligation and correction of the duodenal perforation. The patient recovered well without any complications. Cheng et al.^8^ described a case in which a mycotic abdominal aneurysm was treated by ABFB with infrarenal aortic stump formation. Four months later the patient developed aortic stump rupture which was successfully treated with an iliac occluder stent graft combined with a chimney stent graft for the right renal artery. The patient was treated for six weeks with antibiotics and was discharged. During limited one year follow up, there were no complications. Unfortunately, the patient died due to a hepatocellular carcinoma one year post-operatively. Finally, Hai et al.^9^ treated a thoraco-abdominal aneurysm with a thoraco-iliac bypass with supratruncal aortic stump formation. One hundred and twenty days following primary intervention the patient developed a stump rupture which was treated with a custom made occluder stent graft and iliac to SMA bypass. Follow up was limited to six months in which no complications occurred.

All four cases demonstrate the initial success, effectiveness and technical feasibility of endovascular treatment of aortic stump rupture. All patients were treated successfully, and the plug or coil placement rapidly stopped the bleeding and stabilised the patient. However, follow up after intervention was limited and moreover, in two of the four cases an ADF was present.^7^^,^^8^ These cases are prone to persistent infection, especially when the duodenum is not repaired. The present Case and the case of Marone et al.^7^ suggest that following endovascular repair of an aortic stump rupture, definitive treatment, should be recommended. However, the clinical condition of the patient does not always allow a bridge to surgery approach. In the present case an initial conservative approach was successful for nearly four years.

The use and duration of intravenous antibiotics is a point of discussion. In this Case the patient was treated with selective intravenous antibiotics for a total of six months. PET scan imaging is used as a tool to guide the duration of antibiotic treatment. The exact role of PET imaging and duration of antibiotics remains to be elucidated, especially because, an aorta after surgery usually shows some PET positivity. Finally, although ADF is a rare and serious complication, this case is an incentive to keep providing continuous and tireless care for complex severely ill patients and every surgeon is encouraged not to give up. Positive obstinacy lengthened the survival of the patient for eight years with a reasonably good quality of life.

## Ethics in publishing

For this type of study formal consent is not required.

## Role of the funding source

This study was not supported by any funding.

## Informed consent

Official permission for describing the patients Case was obtained from the patient, in form of a signed informed consent form.

## Conflict of intErest

None.

## References

[1] Zaki M., Tawfick W., Alawy M., ElKassaby M., Hynes N., Sultan S. (2014). Secondary aortoduodenal fistula following endovascular repair of inflammatory abdominal aortic aneurysm due to Streptococcus anginosus infection: a Case report and literature review. Int J Surg Case Rep.

[2] Batt M., Jean-Baptiste E., O'Connor S., Saint-Lebes B., Feugier P., Patra P. (2011). Early and late results of contemporary management of 37 secondary aortaenteric fistulae. Eur J Vasc Endovasc Surg.

[3] Bergqvist D., Björck M. (2009). Secondary arterioenteric fistulation – a systematic literature analysis. Eur J Vasc Endovasc Surg.

[4] Burks J.A., Faries P.L., Gravereaux E.C., Hollier L.H., Marin M.L. (2001). Endovascular repair of bleeding aortoenteric fistulas: a 5-year experience. J Vasc Surg.

[5] Beijer E., Scholtes V.P.W., Moerbeek P., Coveliers H.M.E., Lely R., Hoksbergen A.W.J. (2020). Endovascular treatment of aortic stump blow-out after extra-anatomical repair of aortoduodenal fistula: a Case report and review of literature. CVIR Endovasc.

[6] Terasaki K.K., Allgood M., Finck E.J., Pentecost M.J. (1990). Transcatheter embolization of a ruptured abdominal aortic stump. AJR.

[7] Marone E.M., Mascia D., Kahlberg A., Tshomba Y., Chiesa R. (2012). Emergent endovascular treatment of a bleeding recurrent aortoenteric fistula as a “bridge” to definitive surgical repair. J Vasc Surg.

[8] Cheng M., Lee K.Y., Kwok P.C.H., Fung D.H.S., Cheung M.T. (2013). Endovascular management of aortoduodenal fistula arising from recurrent mycotic aneurysm in an aortic stump. Ann Vasc Surg.

[9] Hai F., Xueming C., Zhe Z., Chenyu L., Bin L., Zhiwen Z. (2014). Treatment of suprarenal abdominal aortic stump rupture using a modified stent graft. Ann Vasc Surg.

