# Supplementary material for: Endovascular Treatment of Aortic Stump Rupture After Extra-anatomic Aortoduodenal Fistula Repair is not a Definitive Treatment: A Case Report and Literature Review
Source: EJVES Vasc Forum. 2022 Mar 30;55:38–41. doi: 10.1016/j.ejvsvf.2022.03.004 (PMC9046796; doi:10.1016/j.ejvsvf.2022.03.004)
Supplement: Multimedia component 1 [file mmc1.docx]

**Supplemental figure 1**

Incisional scar tissue and hernia following multiple abdominal operations

(in color)


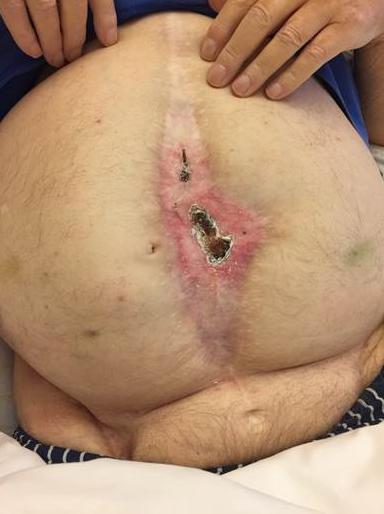


| *Medical past history, chronically* | |
| --- | --- |
| 2005 | Open right-sided hemicolectomy |
| 2006 | Infrarenal abdominal aneurysm: open repair with aortobi-iliac prothesis |
| 2007 | Incisional hernia repair with mesh |
| 2013, march | Duodenal perforation after which the aortic bi-iliac protheses got infected |
| 2013, march 21^st^ | Removal of the infected graft, rifampicin coated Dacron prothesis with omental plasty  Perioperative injury right ureter: suprapubic catheter and urethral splint |
| 2013, march 31^st^ | Duodenal leakage and transfer to our hospital |
| 2013, april 4^th^ | Extra-anatomic right-sided axillo-bifem prothesis, removal of infected graft, aortic stump formation and partial duodenal resection with gastrojejunostomy  Postoperative right-sided nephrostomy due to urethral obstruction |

| **Author** | **Year** | **Intention: definitive ER or bridge** | **Primary tx** | **Duration primary tx - stump rupture** | **ADF** | **Treatment** | **Follow up** | **Complications** |
| --- | --- | --- | --- | --- | --- | --- | --- | --- |
| Terasaki^6^ | 1990 | Definitive ER | Axillobifemoral - infrarenal stump | 50 days | No | Coils / gelatin sponge pledgets | 150 days | No |
| Marone^7^ | 2011 | Bridge | Axillobifemoral - infrarenal stump | 9 years | Yes | Amplatzer Vascular Plug, open repair after 5 days | N/A | No |
| Cheng^8^ | 2013 | Definitive ER | Axillobifemoral - infrarenal stump | 120 days | Yes | Iliac occluder | 1 year | Died after 1 year due to another cause |
| Hai^9^ | 2014 | Definitive ER | Thoracal aorta - iliac bypass - suprarenal stump | 14 days | No | Custom made occluder | 180 days | No |
| Beijer | 2020 | Definitive ER | Axillobifemoral - infrarenal stump | 84 days | Yes | Amplatzer Vascular Plug | 4 years | Infection of aortic stump after 4 years |

**Supplemental table 1**

Overview of the different case reports in which an aortic stump rupture was successfully treated
